# Simultaneous expression of steroid sulfatase and androgen receptor reduced overall survival of patients with epithelial ovarian tumors

**DOI:** 10.1186/s13048-021-00840-x

**Published:** 2021-07-29

**Authors:** Argelia Calvillo-Robledo, Enrique Pedernera, Flavia Morales-Vásquez, Delia Pérez-Montiel, María J. Gómora, Miguel Ángel Almaraz, Paulina García de Alba Graue, Elizabeth Rendón, Horacio Noé López-Basave, Andrés Quintanar-Stephano, Carmen Méndez

**Affiliations:** 1 Departamento de Fisiología, Centro de Ciencias Básicas, Universidad Autónoma de Aguascalientes, Av. Universidad. No. 940, CD. Universitaria, Aguascalientes, AG C.P. 20131 México; 2 Departamento de Embriología y Genética, Facultad de Medicina, Universidad Nacional Autónoma de México, Av. Universidad. 3000, Ciudad de México, C.P. 04510 México; 3 Instituto Nacional de Cancerología, Secretaría de Salud de México, Ciudad de México, México; 4 Hospital Militar de Especialidades de la Mujer y Neonatología, Secretaría de la Defensa Nacional, Ciudad de México, México

**Keywords:** Epithelial ovarian tumor, Ovarian cancer, Steroid sulfatase, Androgens, Androgen receptor

## Abstract

**Background:**

Ovarian cancer is usually diagnosed at an advanced stage due to its early asymptomatic course and late-stage non-specific symptoms. This highlights the importance of researching the molecular mechanisms involved in ovarian carcinogenesis as well as the discovery of novel prognostic markers that could help improve the survival outcome of patients. The aim of this study was to evaluate the expression of the steroid sulfatase (STS) in 154 samples of primary ovarian tumors. This protein is crucial in the intracellular conversion of sulfated steroid hormones to active steroid hormones. The presence of STS, 3β-HSD, and 17β-HSD1 result in the production of testosterone which act through the androgen receptor (AR) in the tumor cell. The presence of STS and AR in epithelial ovarian tumors and their association to the overall survival of patients was evaluated using Kaplan–Meier and Cox regression analyses.

**Results:**

Immunoreactivity for STS was detected in 65% of the tumors and no association was observed with histological subtypes and clinical stages of the tumor. The STS expression in the tumors exhibiting immunoreactive AR resulted in a reduced survival (log-rank test, *p* = 0.032) and a risk factor in univariate and multivariate analysis, HR = 3.46, CI_95%_ 1.00–11.92, *p* = 0.049 and HR = 5.92, CI_95%_ 1.34–26.09, *p* = 0.019, respectively.

**Conclusions:**

These findings suggest that the intracellular synthesis of testosterone acting through its receptor can promote tumor growth and progression. Moreover, the simultaneous expression of STS and AR constitutes an independent predictor of poor prognosis in epithelial ovarian tumors.

## Background

Ovarian cancer (OC) is the eighth most common cancer in women and the second leading cause of death related to gynecological tumors worldwide [1]. Ovarian cancer typically affects postmenopausal women. They are usually diagnosed at an advanced stages of the disease, due to its early asymptomatic course and late-stage non-specific symptoms such as pelvic pain, gastrointestinal symptoms or bloating. In addition, the recurrence of the disease often occurs within 2 years after diagnosis with high resistance to chemotherapy [2].

Epithelial ovarian cancer (EOC) represents more than 90% of diagnosed ovarian tumors and it is a complex and heterogeneous disease at molecular, genetic and histological levels [3]. The main histological subtypes are high grade serous carcinoma (HGSC), low grade serous carcinoma (LGSC), endometrioid, mucinous, and clear cells carcinoma. The epidemiology of EOC is linked to hormonal and reproductive events such as early age at menarche, older age at menopause, and hormone replacement therapy increase the risk of developing EOC, while pregnancy, use of hormone contraception, and prolonged lactation are protective factors [4].

The presence of steroid hormone receptors and their ligands in tumor tissue have been associated to proliferation and epithelium-mesenchymal transition (EMT) in ovarian cancer [5]. Progesterone receptor expression has been previously associated to improved survival outcome in HGSC patients, whereas progesterone and estrogen receptors alone or in combinations result in a best prognosis in patients with endometrioid carcinoma [6]. Moreover, it has been suggested that the enzymes involved in estrogen synthesis could be associated to cell proliferation in several types of cancers, such as endometrial and ovarian cancer [7, 8].

Epidemiological and experimental evidence suggest the involvement of androgens in the development of ovarian tumors [9, 10]. Ose et al. and Trabert et al. observed circulating androgens levels are associated with an increased risk of developing endometrioid and mucinous ovarian carcinoma [11, 12]. However, the role of the androgen receptor in overall survival in EOC patients is still inconclusive, although previous studies have reported that an increased androgen receptor expression is associated with an improved survival [13] other studies have described the absence of any significant association between steroid receptor expression and overall survival [14].

Sulfated steroids like dehydroepiandrosterone sulfate (DHEA-S) from adrenal origin and estrone sulfate (E1-S) are found in high levels in plasma of premenopausal and postmenopausal women [15]. The intracellular presence of the steroid sulfatase (STS) in peripheral tissue allows to obtain active forms of DHEA and E1 which are converted into potent testosterone and 17β-estradiol [16]. The presence and significance of STS has been evaluated in both, normal and cancerous breast tissue [17]. However, the importance of sulfated steroids and steroid sulfatase has been less explored in ovarian cancer and thus the involvement of the enzyme on patient survival remains controversial [18–20].

The goal of the present study is to determine the expression profile of steroid sulfatase (STS), and the androgen receptor (AR) in the serous, endometrioid and mucinous histological subtypes of ovarian tumors in order to provide evidence of their relevance as a risk factor in overall patient survival.

## Results

The histological subtypes of the tumors included in the study were as follows: borderline tumors (BT) 27/154 (18%), low grade serous carcinomas 16/154 (10%), high grade serous carcinomas 44/154 (29%), endometrioid carcinoma 35/154 (23%), mucinous carcinoma 16/154 (10%), clear cells carcinoma 6/154 (4%) and 6% of another tumor subtype. The clinical features of the patients were summarized by the age at diagnosis, the reproductive status, the clinical stages of the tumor according to the FIGO scale, the histological grade registered for endometrioid carcinoma and the success of surgery (Table 1).

**Table 1:** Clinical characteristics by histological subtype in patients with ovarian tumors

	SBT	LGSC	HGSC	Endometrioid	Mucinous	Clear Cells	Others	Total
Median age (years)	39	52	52	49	52	52	50	49
Menopause	8/27 (30)	10/15 (67)	28/42 (67)	20/33 (61)	12/15 (80)	6/6 (100)	5/9 (56)	89/147 (60)
FIGO								
I	15/27 (55)	5/15 (33)	6/42 (14)	17/31 (55)	11/12 (92)	6 /6 (100)	3/8 (38)	63/141 (45)
II	1/27 (4)	2/15 (13)	4/42 (10)	3/31 (10)	–	–	–	10/141 (7)
III	10/27 (37)	6/15 (41)	25/42 (59)	9/31 (29)	1/12 (8)	–	3/8 (38)	54/141 (38)
IV	1/27 (4)	2/15 (13)	7/42 (17)	2/31 (6)	–	–	2/8 (24)	14/141 (10)
Histological Grade								
G1	–	–	–	8/35 (23)	–	–	–	–
G2	–	–	–	22/35 (63)	–	–	–	–
G3	–	–	–	5/35 (14)	–	–	–	–
Surgery debulking								
Optimum	22/24 (92)	10/10 (100)	25/36 (70)	20/27 (74)	13/14 (93)	5/5 (100)	6/8 (75)	101/124 (81)
Suboptimum	2/24 (8)	–	11/36 (30)	7/27 (26)	1/14 (7)	–	2/8 (25)	23/124 (19)

Absolute values (percentage)

*SBT* Serous borderline tumors, *LGSC* Low grade serous carcinoma, *HGSC* High grade serous carcinomas, *G1* Grade 1, *G2* Grade 2, *G3* Grade 3, *FIGO* International Federation of Gynecology and Obstetrics

**Fig. 1:**
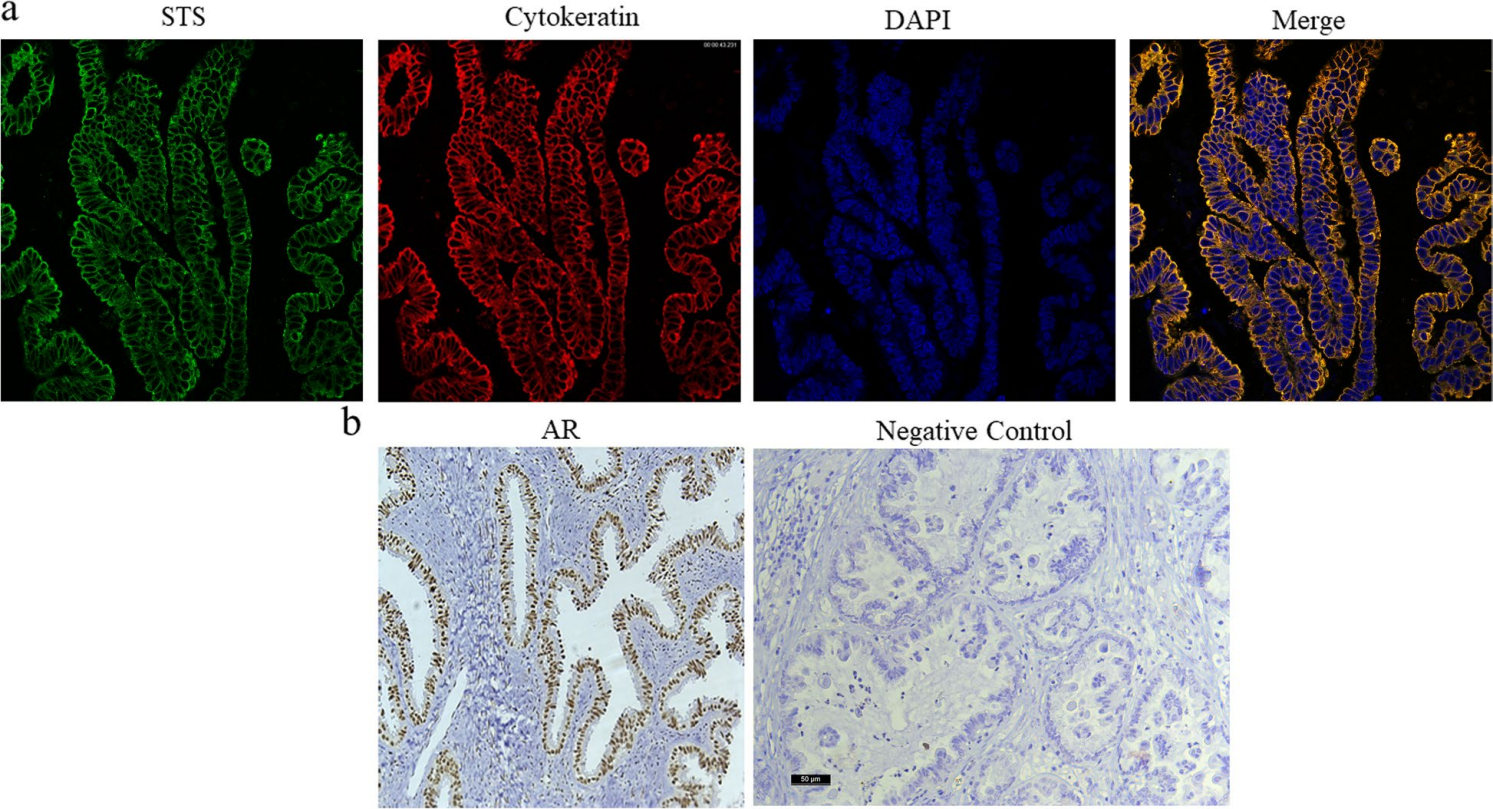
Immunoreactivity for steroid sulfatase (STS) and androgen receptor (AR) in serous ovarian tumor. **A** STS immunofluorescence, the staining localized in cytoplasmic compartment (green), nuclei shown with DAPI (blue) and cytokeratin (red) identifying epithelial cells. **B** AR immunohistochemistry in epithelial tumor cells and negative control. Bar represents 50 µm

Immunofluorescence and immunohistochemistry reactions showed that steroid sulfatase (STS) was located in the cytoplasm of epithelial tumor cells displaying a distribution similar to cytokeratins, whereas the androgen receptor (AR) was identified in the nucleus of tumor epithelium (Fig. 1).

### Frequency of expression of steroid sulfatase and androgen receptor

STS was detected in 65% of the tumors. No association was observed between the expression of STS and the histological subtypes of the tumors (*p* = 0.20) or between the expression of STS and the clinical stage (*p* = 0.84) (Tables 2 and 3).

**Table 2:** Frequency of STS and AR expression by histological subtype

Enzyme or Receptor	SBT	LGSC	HGSC	Endometrioid	Mucinous	*P*-value
STS	17/26 (65)	7/16 (43)	31/44 (70)	21/35 (60)	13/16 (81)	0.203
AR	19/27 (70)	11/16 (69)	26/44 (59)	17/35 (49)	8/16 (50)	0.388

Absolute values (percentage). *P*-value, Chi-square analysis

*STS* Steroid sulfatase, *AR* Androgen receptor, *SBT* Serous borderline tumors, *LGSC* Low grade serous carcinoma, *HGSC* High grade serous carcinoma

**Table 3:** Association between STS and AR expression with the clinical grades of ovarian tumors

Enzyme or Receptor	ECI	ECII	ECIII	ECIV	*P*-value
STS	38/63 (60)	7/10 (70)	34/53 (64)	10/14 (71)	0.838
AR	38/63 (60)	6/10 (60)	30/54 (56)	8/14 (58)	0.962

Absolute values (percentage). *P*-value, Chi-square analysis

*STS* Steroid sulfatase, *AR* Androgen receptor

The androgen receptor was detected in the nucleus of epithelial tumor cells in 58% of the samples. However, there was no association found between the expression of the androgen receptor to the histological subtypes (*p* = 0.39) or the clinical stage (*p* = 0.96). When comparing serous versus non-serous subtypes, we observed a higher frequency of AR expression in serous subtypes (*p*˂ 0.05) (Tables 2 and 3).

### Patients overall survival related to steroid sulfatase and androgen receptor expression

**Fig. 2:**
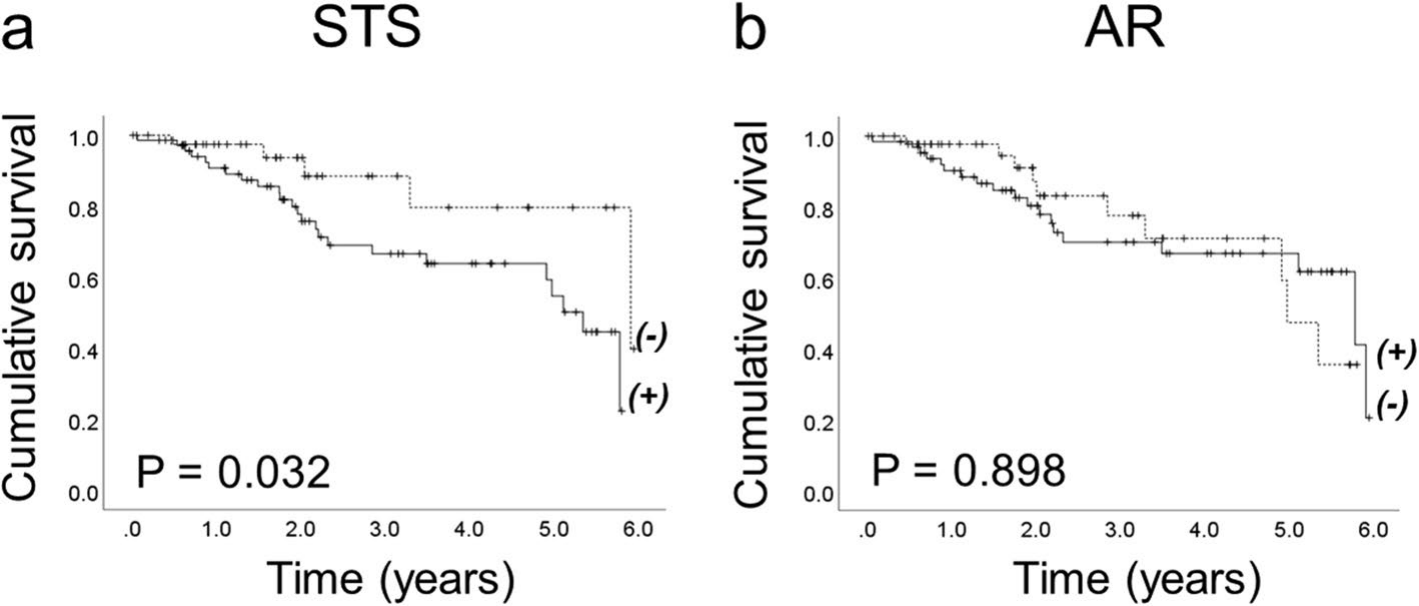
Kaplan–Meier analysis of overall survival of patients associated to the expression of **A** steroid sulfatase and **B** androgen receptor. The survival curve is lower in patients that express STS. *P* < 0.05

The survival curves obtained by Kaplan–Meier analysis displayed in Fig. 2A showed that the presence of STS in epithelial ovarian tumors is related to a reduced survival of patients (log-rank test, *p* = 0.032), whereas the presence of androgen receptor did not modify the patients´ survival during a the 6-years follow-up (Fig. 2B).

Based on Cox proportional hazards regression model the age at diagnosis, the clinical stage (FIGO III and IV), and a suboptimal debulking surgery showed a significant increase in hazard ratio (HR) values in univariate analysis; whereas STS and androgen receptor presence HR values were not significant. In the multivariate analysis, considering age and clinical stage as covariates, HR values for STS and AR remained non-significant, *p* = 0.08 and 0.68, respectively (Table 4).

**Table 4:** Cox proportional regression analysis

Variables	Univariate				Multivariate		
	HR	95% CI	*p*-value		HR	95% CI	*p*-value
STS	2.032	0.83–4.96	0.119		2.435	0.91–6.48	0.075
AR	1.126	0.53–2.35	0.753		1.170	0.55–2.48	0.681
Age	1.035	1.00–1.06	**0.009**		1.025	0.99–1.05	0.105
FIGO							
I	*Reference*						
II	3.551	0.32–2.93	0.301		3.330	0.30–36.79	0.326
III	12.785	2.93–56.72	** < 0.001**		12.267	2.79–53.84	** < 0.001**
IV	26.390	5.69–122.3	** < 0.001**		20.102	4.24–95.22	** < 0.001**
Surgery	5.015	2.16–11.64	** < 0.001**		–	–	–

*HR* Hazard ratio, *STS* Steroid sulfatase, *AR* Androgen receptor, *FIGO* International Federation of Gynecology and Obstetrics

### Co-expression of steroid sulfatase and androgen receptor on patient overall survival

**Fig. 3:**
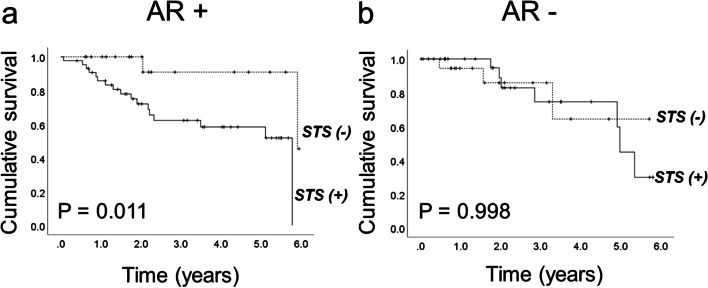
Kaplan–Meier analysis of overall survival in patients according to the presence of steroid sulfatase in the tumor. The population is stratified into two groups: **A** positive expression of androgen receptor and **B** non-expression of androgen receptor. The population of patients expressing androgen receptor and sulfatase steroid, demonstrate poor survival. *P* < 0.05

The total of the patients included in the study were stratified considering the presence or absence of AR in the ovarian tumor evaluating their overall survival. The survival curves demonstrated that the presence of STS represents a worse prognosis for the patients with AR positive tumors (*p* = 0.011); whereas in AR negative tumors, STS expression did not modify the overall survival of patients (*p* = 0.998) (Fig. 3). The proportional hazard regression analysis displayed the following values for STS in AR positive tumors: HR = 3.46, 95%CI 1.00–11.92, *p* = 0.049 and HR = 5.92, 95%CI 1.34–26.09, *p* = 0.019 univariate and multivariate analysis, respectively. The HR values in AR negative tumors were not significant (Table 5).

**Table 5:** Cox regression analysis of steroid sulfatase based on the presence of androgen receptor

Expression	Univariate			Multivariate		
	HR	95% CI	*p*-value	HR	95% CI	*p*-value
STS /AR (+)	3.463	1.00–11.92	0.049	5.917	1.34–26.09	0.019
STS /AR (-)	0.766	0.19–2.94	0.697	1.033	0.19–5.49	0.970

*HR* Hazard ratio, *STS* Steroid sulfatase, *AR* Androgen receptor

## Discussion

The present retrospective study demonstrates that the simultaneous expression of STS and AR in epithelial ovarian tumors reduced overall survival of the patients. This information was obtained from tumor samples collected at the diagnostic surgery in patients who had not undergone neoadjuvant therapy. The presence of STS and AR was demonstrated following an immunohistochemistry and immunofluorescence protocol and represents an accessible and relatively inexpensive methodology to characterize the tumor.

Results reported herein demonstrate that STS was present in 68% of the ovarian tumors, a frequency similar to that previously described in clear cells ovarian carcinoma [18], with the enzyme being localized in the cytoplasm of epithelial tumor cell, as previously shown by immunohistochemistry in epithelial ovarian cancer [18, 20]. Likewise, sulfatase activity has been demonstrated in tumors of the female reproductive system and corroborated in ovarian cancer samples by the incubation of tissue homogenates with estrone sulfate isotopes by evaluating estrone and 17β-estradiol formation [19, 21].

The presence of STS was not associated with the FIGO stage of the tumor, and a similar finding was demonstrated when comparing to the clinical stage in clear cells ovarian carcinoma [18]. The relationship of STS to the histological subtype of ovarian tumor was not statistically significant in the present study. Variations between subtypes should be considered in subsequent extensive studies of epithelial ovarian tumors.

Overall survival curves obtained through Kaplan–Meier analysis displayed a poor survival rate in STS positive tumors, however, this finding was not corroborated by a strong analysis like Cox proportional hazard regression. A previous study reported that patients with a tumor tissue displaying high sulfatase activity had a reduction in progression-free survival but the overall survival remained unchanged [18]. A similar absence of overall survival reduction was described by Mungenast et al. [20] in ovarian cancer. Further studies are needed in order to demonstrate that survival of patients could be affected by the expression of STS in ovarian tumors.

In the present study, a worse overall survival related to STS expression was demonstrated by Kaplan–Meier and Cox regression analyses in patients with AR positive tumor expression, relevant findings which could be explained by the intracellular formation of androstenedione and testosterone from DHEA-S in peripheral tissues [22]. High level of sulfated DHEA has been shown in the plasma of pre and postmenopausal women as well as STS, 3β-HSD and 17β-HSD1 have been described in ovarian tumors [23], suggesting that active androgens acting via their receptor can promote carcinogenesis [24]. Alternatively, DHEA-S via aromatase pathway and estrone-sulfate are transformed to estrone and 17β-estradiol which favor the tumor growth [25]. Another possibility to be considered is the combined action of both androgens and estrogens in ovarian tumors, through which the presence of receptors and their active ligands will stimulate, angiogenesis, cell proliferation, epithelial-mesenchymal transition, migration, invasion, and metastasis, acting directly or through growth factors and cytokines.

## Conclusions

In conclusion, our results demonstrate that the simultaneous expression of steroid sulfatase and the androgen receptor at the time of diagnosis of epithelial ovarian tumors reduced overall patient survival and constitutes an independent prognostic factor that maybe taken into consideration when conducting the clinical evaluation and treatment plan of patients. While, further knowledge about the role of these proteins in ovarian tumors is needed, the results obtained in the present study constitute a solid base of significant data for the use in targeted treatments of patients diagnosed with this malignancy.

## Methods

### Samples and patients

The study group comprised 154 samples of patients with pathological diagnosis of primary ovarian tumor from the Military Hospital for Women’s Specialties and Neonatology, SEDENA (Mexico’s Defense Ministry), and the National Institute of Cancerology in Mexico City spanning a time period of 10 years between 2008 and 2018. Written consent was obtained from each patient in order to participate in this study, none of whom had received chemotherapy or radiotherapy prior to surgery, per inclusion criteria. The tissue processing was done in accordance with the protocols established for international tumor banks and was approved by the Ethics Committee of the School of Medicine of the National Autonomous University of Mexico (UNAM-108/2015) as well as the Military Hospital of Women’s Specialties and Neonatology (310–18) and the National Institute of Cancerology (INCan) (019/060/OMI).

### Immunohistochemistry

The study samples were obtained through intraoperative biopsy for diagnosis by the pathology department, after which whole sections of paraformaldehyde-fixed and paraffin-embedded tissue samples were serial sectioned at 3 μm thickness and placed on coated glass slides (Biocare Medical, Pacheco, CA, USA) which were deparaffinized by incubation in xylol and rehydrated through graded concentrations of ethanol, with the antigens being retrieved with Diva Decloaker citrate buffer (Biocare Medical) in a pressurized cooker at 110 °C for 15 min. Endogenous peroxidase was blocked by hydrogen peroxide at 1% H_2_O_2_ for 10 min. (Biocare Medical) after rising in PBS (pH 7.3) incubating the slides overnight at 4 °C with the following polyclonal rabbit primary antibody to AR, diluted 1:50 (Sc816, Santa Cruz Biotechnology, Santa Cruz, CA), using Mach2 anti-rabbit HRP as a secondary antibody for 1 h at room temperature (Biocare Medical) and signal detection was achieved using a diaminobenzidine chromogen kit (DAB) (Biocare Medical). The negative controls were tissue samples in which the primary antibody was substituted with PBS including the positive control tissues (placenta for STS and mice testis for AR) as well in each immune reaction, rinsing the slides with water after counterstaining them with hematoxylin. To assess the positivity ratio of reactions we evaluated the intensity of the staining and the percentage of labeled cells in the tissue samples. Thus the samples with no noticeable staining were rated as 0, while cells exhibiting a light staining were labeled as 1, cells with a moderate staining were marked as 2, and cells with a strong staining were designated as 3. The percentage of labeled cells was considered as: 1 = 10–25%, 2 = 26–50%, 3 = 51–80%, and 4 ˃ 80%. Positive reaction was considered whenever the index obtained from multiplying intensity and percentage was ≥ 2. Sample analyses were performed by three independent observers (MJG, MAA, and EP).

### Immunofluorescence

Slides were incubated overnight at room temperature with the following polyclonal rabbit primary antibodies: anti-STS, diluted 1:200 (GTX105498, GeneTex, Inc.) and further incubated with a secondary antibody goat-anti-rabbit Alexa 488, diluted 1:1000 (Thermo Fisher Scientific, Waltham. MA, USA). In order to identify epithelial cells, the slides were secondarily incubated overnight at 4 °C, with mouse monoclonal anti pan-cytokeratin AE1/AE3 + 8/18, diluted 1:100 (CM162C, Biocare Medical). The samples were washed with PBS and incubated with the secondary antibody Alexa Fluor 647 donkey anti-mouse, diluted 1:500 (A31571, Thermo Fisher Scientific). Placenta was used as a positive control in each immune reaction. The nucleus was stained with 4′, 6-Diamidino-2-Phenylindole, Dihydrochloride (DAPI) (Sigma-Aldrich, Inc). The slides were mounted with VectaShield mounting medium (Vector Laboratories) and the immunolabeled slices were observed by confocal laser microscope Leica TCS SP5 (Leica Microsystems, Wetzlar, Germany) using excitation spectral laser lines at 488 and 647 nm.

### Statistical analysis

Association between variables was analyzed by Pearson’s Chi square, to determine the hazard ratio considering every possible variable we performed multivariate analysis using Cox regression model and calculated the overall survival rate through Kaplan–Meier analysis evaluating all significant data using SPSS 21.0 statistical software. Differences were considered significant at *p* < 0.05.

## Data Availability

The dataset used and/or analyzed during the current study are available from the corresponding authors on reasonable request.
